# Activation and targeting of ATG8 protein lipidation

**DOI:** 10.1038/s41421-020-0155-1

**Published:** 2020-05-05

**Authors:** Sascha Martens, Dorotea Fracchiolla

**Affiliations:** Department of Biochemistry and Cell Biology, Max Perutz Labs, University of Vienna, Vienna BioCenter, Dr. Bohr-Gasse 9/5, 1030 Vienna, Austria

**Keywords:** Macroautophagy, Phagocytosis

## Abstract

ATG8 family proteins are evolutionary conserved ubiquitin-like modifiers, which become attached to the headgroup of the membrane lipid phosphatidylethanolamine in a process referred to as lipidation. This reaction is carried out analogous to the conjugation of ubiquitin to its target proteins, involving the E1-like ATG7, the E2-like ATG3 and the E3-like ATG12–ATG5–ATG16 complex, which determines the site of lipidation. ATG8 lipidation is a hallmark of autophagy where these proteins are involved in autophagosome formation, the fusion of autophagosomes with lysosomes and cargo selection. However, it has become evident that ATG8 lipidation also occurs in processes that are not directly related to autophagy. Here we discuss recent insights into the targeting of ATG8 lipidation in autophagy and other pathways with special emphasis on the recruitment and activation of the E3-like complex.

## Introduction

The Atg8 protein is a ubiquitin-like protein, which was identified in *S. cerevisiae* in the course of screens designed to discover genes required for the process of macroautophagy (hereafter referred to as autophagy) or the related cytoplasm-to-vacuole targeting (Cvt) pathway^1–3^. Subsequent sequence analysis revealed homology of Atg8 to the mammalian LC3 and GABARAP proteins^4–6^. In total the human genome contains seven functional ATG8 genes (LC3A, LC3B, LC3B2, LC3C, GABARAP, GABARAPL1, and GABARAPL2)^7^, of which LC3B2 appears to be expressed at very low levels^8^. The genome of many plants codes for even more ATG8 proteins. For example, *Arabidopsis thaliana* has 9 ATG8 genes^9^. Structural studies revealed that the core of the ATG8 structure consists of an ubiquitin-like fold^10^. However, unlike ubiquitin, which is conjugated to the lysine residues of target proteins via an isopeptide bond involving its C-terminal glycine residue, ATG8 proteins become attached to the amino headgroup of membrane lipids^11^. In vivo the main target of this conjugation, which is also referred to as lipidation, is phosphatidylethanolamine (PE), although at least in vitro phosphatidylserine can also serve as substrate^12–14^. Because the process of autophagy can be conveniently traced employing ATG8 proteins as markers, they have been widely used to monitor and study the pathway^15,16^. In this review, we will summarize how ATG8 proteins function in autophagy. It is becoming increasingly clear that there are roles for these proteins in pathways that are not strictly related to autophagy and we will also summarize the current knowledge about the association of ATG8s with these pathways. Finally, we will discuss how the ATG8 conjugation machinery is activated and targeted to the correct membrane with special emphasis on the E3-like ATG12–ATG5–ATG16 complex.

### The function of ATG8 proteins in autophagy

**Fig. 1:**
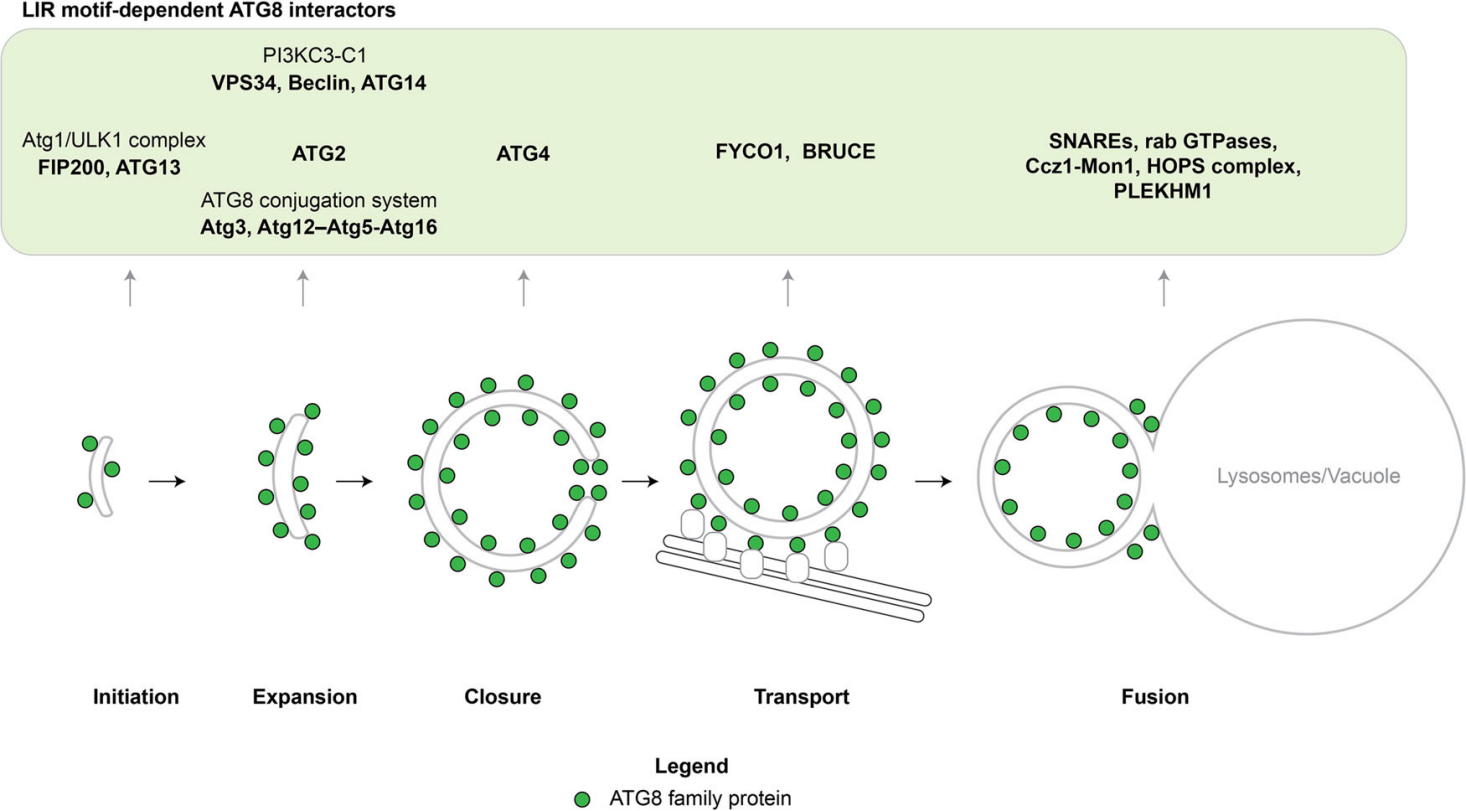
LIR motif-dependent ATG8 interactions. During the process of macroautophagy, ATG8 family proteins participate in autophagosome formation at different stages from initiation to the fusion with the lytic compartment. These interactions are typically mediated by LIR motifs in the target proteins. In the scheme, the ATG8 interactors found in yeast and humans are shown. The ATG8 protein family members are depicted in green.

Autophagy mediates the delivery of various cytoplasmic substances into lysosomes (the vacuole in yeast) for degradation. This is achieved by the sequestration of this material referred to as cargo within double membrane vesicles named autophagosomes (Fig. 1). Autophagosomes are generated de novo and first appear as small membrane structures called isolation membranes or phagophores. The isolation membranes capture the cargo as they grow. Subsequently, the isolation membranes close to give rise to autophagosomes, which eventually fuse with a lysosome wherein the inner membrane and the cargo are finally degraded^17^.

The formation of autophagosomes depends on a number of highly conserved factors that are collectively referred to as the autophagy machinery and that can be subdivided into distinct functional modules. According to the guidelines, we will refer to the human proteins with all capital letters (ATG) and to the *S. cerevisiae* proteins with only the first letter in capital (Atg; Table 1). In particular, these are (1) the ULK1/2 kinase complex composed of the ULK1/2 protein kinase, the FIP200 scaffold protein, ATG13 and ATG101, (2) vesicles containing the ATG9A protein, (3) the class III phosphatidylinositol 3-phosphate kinase complex 1 (PI3KC3-C1) composed of the VPS34 lipid kinase, VPS15, Beclin and ATG14, (4) the WIPIs and ATG2, and (5) the ATG8 conjugation system including the ATG12–ATG5–ATG16L1 complex^18–20^. Humans have six ATG8 proteins that are expressed at considerable levels and that can be subdivided into the LC3 (LC3A, LC3B, LC3C) and GABARAP (GABARAP, GABARAPL1, GABARAPL2) families. In their entirety we will refer to them as ATG8 proteins unless a particular family member is mentioned.

**Table 1:** Atg/ATG nomenclature.

Species Protein group	*S. cerevisiae* (Atg)	*H. sapiens* (ATG)
Protein kinase complex	Atg1, Atg13, Atg11/Atg17, Atg29, Atg31	ULK1/2, ATG13, FIP200, ATG101
Vesicles	Atg9	ATG9A
Lipid kinase complex	Vps34, Vps15, Atg6, Atg14	VPS34, VPS15, Beclin, ATG14
PI3P sensors and lipid transfer	Atg21, Atg18, Hsv2, Atg2	WIPI1, WIPI2, WIPI3, WIPI4, ATG2
Ubiquitin-like conjugation machineries	Atg7, Atg10, Atg5, Atg12, Atg16, Atg3, Atg8, Atg4	ATG7, ATG10, ATG5, ATG12, ATG16L1, ATG3, LC3s/GABARAPs, ATG4A/B/C/D

**Fig. 2:**
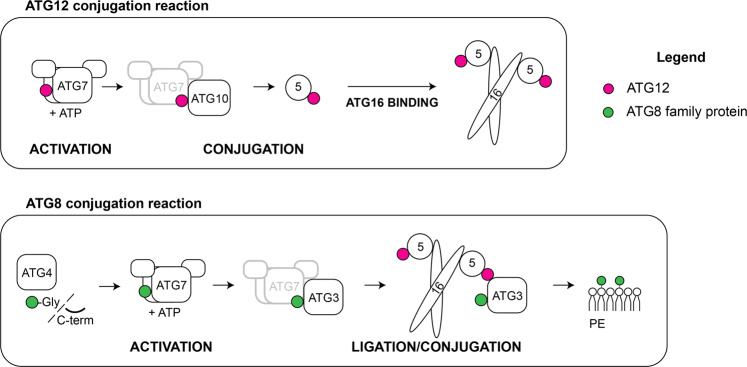
The ATG12 and ATG8 ubiquitin-like conjugation systems. Similar to the classical ubiquitination cascades, the ubiquitin-like proteins ATG12 and ATG8 undergo a series of enzymatic reactions. ATG8 is first modified by the ATG4 protease and, likewise for ATG12, is activated by the E1-like enzyme ATG7 with consumption of ATP (ACTIVATION). ATG8 and ATG12 are then conjugated to the target protein (ATG5) or lipid (Phosphatidylethanolamine, PE) by the cognate E2-like enzymes (ATG10 and ATG3) (CONJUGATION). The last step of ATG8 lipidation is stimulated by the E3-like ligase ATG12–ATG5–ATG16 complex (LIGATION), while the ATG12 conjugation system lacks a known enzyme for the E3-like ligase activity. The ATG12–ATG5 conjugate interacts with the coiled-coil protein ATG16 via the ATG5 subunit, and further assembles into a hexamer. Legend: the ATG8 protein family members are indicated in green and the ATG12 proteins are indicated in magenta.

The lipidation of ATG8 proteins occurs analogous to the conjugation of ubiquitin to target proteins (Fig. 2)^11,21^. First the C-terminus of the ATG8 proteins becomes proteolytically cleaved by ATG4 family proteases to expose a glycine residue^22^. ATG8 is subsequently transferred to a cysteine residue in the E1-like ATG7 under the consumption of ATP. From there ATG8 is transferred to the E2-like ATG3, which mediates the attachment of ATG8 via its C-terminal glycine to the headgroup of PE. This last step is promoted by the ATG12–ATG5–ATG16 complex, which acts in an E3-like manner and which is itself the product of a ubiquitin-like conjugation reaction (Fig. 2)^12,23,24^. To form the complex, the ubiquitin-like ATG12 is activated by ATG7, transferred to the E2-like ATG10 and from there to an internal lysine residue of ATG5. The ATG12–ATG5 conjugate subsequently associates with the dimeric coiled-coil protein ATG16^25–27^. Although in vitro ATG8 conjugation can occur in the absence of the E3-like complex, its presence vastly accelerates the reaction and it is essential for ATG8 lipidation in cells^12,23,25–27^. The attachment of ATG8 proteins to PE is reversible as the ATG4 proteases can remove them from the membrane^28,29^.

The targeting and activation of ATG8 lipidation are genetically the most downstream events within the cascade of the core autophagy machinery depending on the presence of the upstream acting factors^30,31^. A crucial event is the recruitment of the E3-like complex. In autophagy the main mechanism for its recruitment is via the PI3KC3-C1-WIPI2 axis, where the PI3P generated by the PI3KC3-C1 recruits the WIPI2 proteins, which in turn recruit and activate the ATG12–ATG5–ATG16L1 complex and thus ATG8 lipidation^32–34^. Other mechanisms to recruit the E3 to the site of autophagosome biogenesis via the ULK1/2 kinase complex^35–37^, membrane binding^23,38,39^ and cargo receptor interaction^40^ do also exist and will be discussed in detail below.

The functions of the ATG8 proteins in autophagy are manifold and even though their lipidation is a downstream event they can play important roles in the recruitment of upstream components of the autophagy machinery via feedback loops^41^. Mechanistically, most of the many roles of ATG8 in autophagy can be attributed to their functions as binding platforms for other proteins^42,43^, in particular once they are attached to the membrane^44^. These interactions are generally, but not exclusively^45,46^, mediated by so-called LC3-interacting regions (LIRs, also referred to as ATG8-intercating motifs (AIMs) or LC3 recognition sequence (LRS))^47–49^. These LIR motifs, which are typically composed of two hydrophobic amino acids spaced by two random residues and preceded by negatively charged residues are located in unstructured regions of the ATG8-interacting proteins. While it is possible to predict LIR motifs with considerable confidence, the variations of these motifs necessitate their experimental confirmation^50,51-53^. The two hydrophobic residues within the LIR motifs bind to two hydrophobic pockets in the ATG8s^54^. The affinities of the LIR motifs for the ATG8 proteins are generally moderate with K_d_s in the low µM range^55^. However, due to the fact that the ATG8 proteins become highly concentrated on the nascent autophagosomal membrane in the course of their conjugation, these membranes efficiently attract proteins harboring LIR motifs^44,56^. Many core autophagy factors including the ULK1/Atg1 kinase, FIP200, ATG13, VPS34, Beclin, ATG14, ATG2, ATG3, ATG4 and the Atg12–Atg5–Atg16 complex contain LIR motifs^57–64^ (reviewed in^52^). The autophagy machinery thereby generates a positive feedback loop by first recruiting and activating the ATG8 lipidation machinery, which in turn serves to recruit further upstream factors (Fig. 2). However, the ATG4 proteins, which catalyze the reverse reaction i.e. ATG8 delipidation are also recruited to the membrane via their interaction with ATG8^62,63^. Presumably the activity of the ATG4 proteins is inhibited by the activity of the ULK1/2 kinase before autophagosomes are completed^65^.

In addition to the core autophagy machinery, ATG8 proteins recruit factors to the completed autophagosome, which are important for the trafficking and fusion with lysosomes (Fig. 1)^66,67^. The ATG8 proteins of the GABARAP subfamily are particularly important in this respect in human cells^68^. Among the factors recruited are PLEKHM1^69^, SNAREs^70,71^, FYCO1^72^, BRUCE^73^, the HOPS complex^74^, the Mon1–Ccz1 complex^75^. In combination, these proteins and protein complexes mediate the recruitment of molecular motors that bring the completed autophagosomes in proximity to lysosomes, recruit and activate Rab GTPases, aid the tethering of the autophagosomes to the lysosomal membrane and recruit SNARE proteins that execute the final membrane fusion event (Fig. 1) (reviewed in^66,67,76^).

Apart from serving as docking platforms, ATG8 proteins have intrinsic membrane tethering and remodeling activities, which may aid the closure of isolation membranes to form autophagosomes and fusion with lysosomes. When concentrated on membranes, *S. cerevisiae* Atg8, *C. elegans* LGG-1 and LGG-2 as well as human LC3B, GABARAP, and GABARAPL2 efficiently tether small and giant unilamellar vesicles in vitro^39,77–81^. At least for Atg8 and LC3B, membrane fusion required high PE concentrations^82^. Yeast Atg8 was also reported to induce membrane curvature^83^. Furthermore, ATG8s were observed to mediate either hemifusion, a state where only the two contacting monolayers of the two opposing membranes fuse and thus no content mixing occurs^84^, or full fusion^77–79^. The membrane remodeling activities were attributed to the two N-terminal helices, which represent ATG8 unique extension of the ubiquitin fold and which are only loosely packed to the core of the globular proteins^85^. The loose packing of the N-terminus is particularly important for the fusogenic properties of the proteins^79^.

In summary, ATG8 proteins play a plethora of roles during the biogenesis of autophagosomes starting from early stages after autophagosome nucleation, via isolation membrane expansion, its closure, the transport of autophagosomes along microtubules to lysosomes until the final fusion event (Fig. 1). In addition, the ATG8 conjugation machinery is important to render the inner autophagosomal membrane an efficient target for lysosomal hydrolases^86^.

While autophagy was initially thought to be largely non-selective, it has become clear that the process can be highly specific with regard to the cargo that is captured by autophagosomes. This is particularly true for autophagosomes that are formed in the absence of starvation, when autophagy is induced by the presence of specific cargo and not by mTOR inactivation^87^. It is beyond the scope of this review to list all known selective autophagy pathways that degrade among other materials damaged mitochondria, bacterial pathogens and aggregated proteins^88–91^. The basis for selectivity in autophagy is at least in part mediated by the tethering of the cargo to the nascent autophagosomal membrane. This mechanism acts in conjunction with the local activation of autophagosome formation at the cargo^40,92–97^. Most cargoes do not directly bind the membrane but are linked to it by cargo receptors, which simultaneously bind the cargo and ATG8 proteins that decorate the membrane. A plethora of cargo receptors has been identified. The most extensively studied ones are the yeast Atg19 as well as the human p62/SQSTM1, NDP52, Optineurin and NBR1 proteins. These proteins are soluble and are recruited to the cargo on demand. There is also a growing number of adapter proteins that are embedded in the membrane of organelles, most notably the endoplasmic reticulum, and which have the ability to bind to ATG8 proteins. A comprehensive list of cargo receptors and their discussion is provided in the following reviews^52,66,90^. Many cargo receptors are dimeric or multimeric, and some of them have more than one LIR motif^56,98–102^, resulting in a high avidity interaction with ATG8 proteins when they are concentrated on the membrane^44,56^. ATG8-decorated membranes are therefore highly efficient recruiters of proteins, protein complexes and polymers that contain multiple LIR motifs.

A certain degree of functional specialization among the human ATG8 proteins does exist^21,103^. For example, autophagosome formation and their degradation triggered by starvation and by PINK1/Parkin proceeds normally in HeLa cells lacking LC3 proteins, while GABARAP proteins are important for the fusion of autophagosomes with lysosomes^68,104^. This defect in fusion was attributed to reduced recruitment of PLEKHM1 to completed autophagosomes^68^. PLEKHM1 is a protein linking ATG8 positive membranes with Rab7 on lysosomes and the HOPS complex^69^. It was shown that its ATG8-interacting motif binds preferentially to GABARAP as opposed to LC3B via a GABARAP interaction motif (GIM), which is a variant of the canonical LIR motif^105^. In *C. elegans*, the GABARAP-like LGG-2 is more important for autophagosome formation and their fusion with lysosomes than the LC3-like LGG-1^74,79^. Thus, current evidence points to a more important role of the GABARAP proteins in autophagy compared to the LC3 subfamily. However, the LC3 proteins may have crucial roles in selective autophagy pathways. For example, the degradation of p62 upon starvation-induced autophagy depends on the LC3 and GABARAP proteins^68,106,107^. LC3C is special among the ATG8 proteins as it binds to a noncanonical LIR motif termed CLIR for LC3C-interacting region, which is present in the autophagy receptor NDP52. LC3C and a functional CLIR motif were shown to be important for antibacterial autophagy^108^.

Given the many roles of the ATG8 proteins during autophagosome formation and cargo capture it is surprising that at least in some mammalian cells the presence of these proteins and their conjugation is not essential for autophagosome formation and the selective capture of mitochondria in PINK1/Parkin induced mitophagy^68,86^. This is in line with the fact that knockout mice lacking the ATG8 conjugation machinery are born viable^109–112^, while mice lacking components of the upstream machinery tend to have more severe phenotypes^113^. In contrast, in yeast Atg8 and its lipidation are essential for autophagosome formation^16^. How can the discrepancy between the finding that ATG8 proteins are defining features of autophagic membranes also in mammalian cells, that attract the autophagy machinery and cargo receptors be reconciled with their non-essentiality? First, the lipidation of ATG8 proteins is the most downstream step among the events occurring after initiation of autophagy^30,31^. Thus, autophagosomes are likely nucleated in the absence of ATG8 lipidation even in wild type cells. Redundant mechanisms for the recruitment of further autophagy factors during the expansion of isolation membranes must therefore exist in form of protein–lipid and protein–protein interactions. With regard to selective autophagy, ATG8s are indeed essential for the degradation of p62 upon starvation^68^. In PINK1/Parkin the induction of autophagosome formation occurs in vicinity of the mitochondria via the recruitment of FIP200 and the ULK1/2 complex by NDP52^95^. Therefore, in this and perhaps other forms of cargo-induced selective autophagy^94,96,97^ where the membrane is already in vicinity of the cargo from the beginning of the process the interaction of cargo receptors with ATG8 proteins might not be required to engulf the cargo. Instead, other proteins present on the cargo might link the cargo to the membrane. For example, cargo receptors bind components of the autophagy machinery, which in turn bind the membrane^94–97,114^. Regardless, the fact that the ATG8 proteins and their conjugation is not essential for autophagosome biogenesis and for the selection of certain cargoes does not mean that they are not important factors during these processes as most forms of macroautophagy are associated with ATG8 lipidation. It appears that the pathway is robust enough, such that it can proceed even in the absence of some core factors, albeit in a less efficient manner.

### ATG8 conjugation to single membranes

For a long time, the process of ATG8 protein lipidation was considered a hallmark of autophagy that is exclusively associated with autophagosomes or their precursors. Indeed, many assays to follow the process of autophagy are based on the fact that these proteins are covalently coupled to these membranes and that a fraction of these proteins, which is present on the inner autophagosomal membrane is finally degraded within lysosomes^115,116^. However, it has become evident that ATG8 proteins are also present on membranes that are not associated with autophagosomes. The conjugation of ATG8 proteins to single membranes was first reported for a process called entosis, wherein one cell takes up another cell by a special form of phagocytosis, and for micropinocytosis^117^. Since then many endocytic events have been reported to be accompanied by ATG8 protein lipidation and they are commonly referred to as LC3-ascociated phagocytosis (LAP). ATG8 conjugation in LAP is independent of the ULK1/2 complex and ATG9 but requires the conjugation machinery including ATG5, ATG7 and ATG16, ROS production, and at least under certain conditions the activity of the PI3KC3 but not ATG14^118–125^. The function of ATG8 proteins on the endocytic organelles appears to be the acceleration of cargo degradation^117^. In addition to the plasma membrane-derived phagocytic and endocytic structures, ATG8 proteins have also been observed on endosomal and lysosomal membranes^126,127^. The distinction between endocytic and endo-lysosomal membranes is not always trivial as the membranes of endocytic vesicles and phagosomes fuse with endosomes and lysosomes. Agents that cause membrane damage are sufficient to elicit ATG8 lipidation on endo-lysosomal membranes suggesting that membrane defects are activators of the conjugation machinery^126,127^. The ROS produced during LAP may have a similar effect by oxidizing lipids and thus altering the membrane structure^128^. ATG8 protein conjugation has also been associated with the autophagic degradation of damaged endo-lysosomal structures by lysophagy^129,130^. Some of the ATG8 protein lipidation observed in lysophagy may actually correspond to their direct lipidation to the lysosomal membrane. Furthermore, it was demonstrated that ATG8 conjugation can also occur on the plasma membrane upon its damage by pathogen-derived factors and further that the presence of the lipidated ATG8 proteins aids plasma membrane repair^131^. ATG8 protein conjugation was also shown to be involved in various forms of non-conventional secretion including the release of exosomes and cytokines^132–140^.

A further non-autophagic function has been described for LC3C, which promotes COPII-dependent ER export via its interaction with TECPR2^141^. This is somewhat reminiscent for the intra-Golgi trafficking functions reported for GABARAP and GABARAPL2 (also known as GATE-16)^6,142,143^.

In summary, ATG8 proteins and their lipidation machinery have various functions that are not directly related to autophagy. Therefore, care needs to be taken when interpreting phenotypes of knockout studies and when following the production of lipidated ATG8 proteins as measures for autophagic activity.

### Mechanisms regulating the targeting and activation of ATG8 lipidation

Given the many roles of the ATG8 proteins in various pathways, the question arises how their conjugation is targeted and activated at the right time and place. All conjugation events appear to be dependent on the ATG7/ATG3/ATG12–ATG5-ATG16 cascade and thus analogous to classical ubiquitination reactions requiring the activities of an E1, E2, and E3 (Fig. 1)^21,144^. The protein, which transfers ATG8 to PE is the E2-like ATG3. In vivo, yeast Atg3 has been localized together with Atg8 at the PAS and on the growing isolation membrane^145^. In vitro, the presence of ATG7 and ATG3 is sufficient for lipidation to occur on small unilamellar vesicles (SUVs) containing a high percentage of PE.

However, the activity of ATG3 is vastly stimulated by the ATG12–ATG5–ATG16 complex in vitro and all subunits of this protein complex are essential for detectable ATG8 conjugation in cells^12^. For the yeast conjugation system Atg16 is not required for the E3 activity of the complex in vitro, while for the mammalian system it is important, showing differences in the mechanism of action between the yeast and mammalian E3-like complex^12,23,34,39^.

Structural studies of the yeast and human conjugation machineries showed that ATG8 proteins are first bound by the extreme C-terminal domain (ECTD) of ATG7 through hydrophobic and aromatic residues that insert deeply into the two hydrophobic pockets of the ubiquitin-like fold, in a mode similar to that described for LIR motifs^146^. Subsequently, the substrates are transferred to the adenylation site (AD) of the ATG7 N-terminal domain containing the catalytic cysteine. ATG7 is a homodimer and binds the cognate E2s followed by conformational changes allowing the juxtaposition of the active site in the E2 with that of the E1. Thereby the substrate is transferred from ATG7 to ATG3^146–148^. The actual lipidation reaction is promoted by the ATG12–ATG5–ATG16 complex. The interaction of the complex and ATG3 is mediated by a patch in ATG12 and a loop in ATG3^149^. Details of the mechanisms of the E2 activation by the E3-like complex enzyme have been recently further elucidated. The so-called E123IR (E1-2-3 Interacting Region) in yeast Atg3 acts as an allosteric switch that initially interacts intra-molecularly locking the protein in an inactive state. Upon interaction with the Atg12–Atg5–Atg16 complex his inhibition is released allowing ATG8 attachment to PE^150^.

**Fig. 3:**
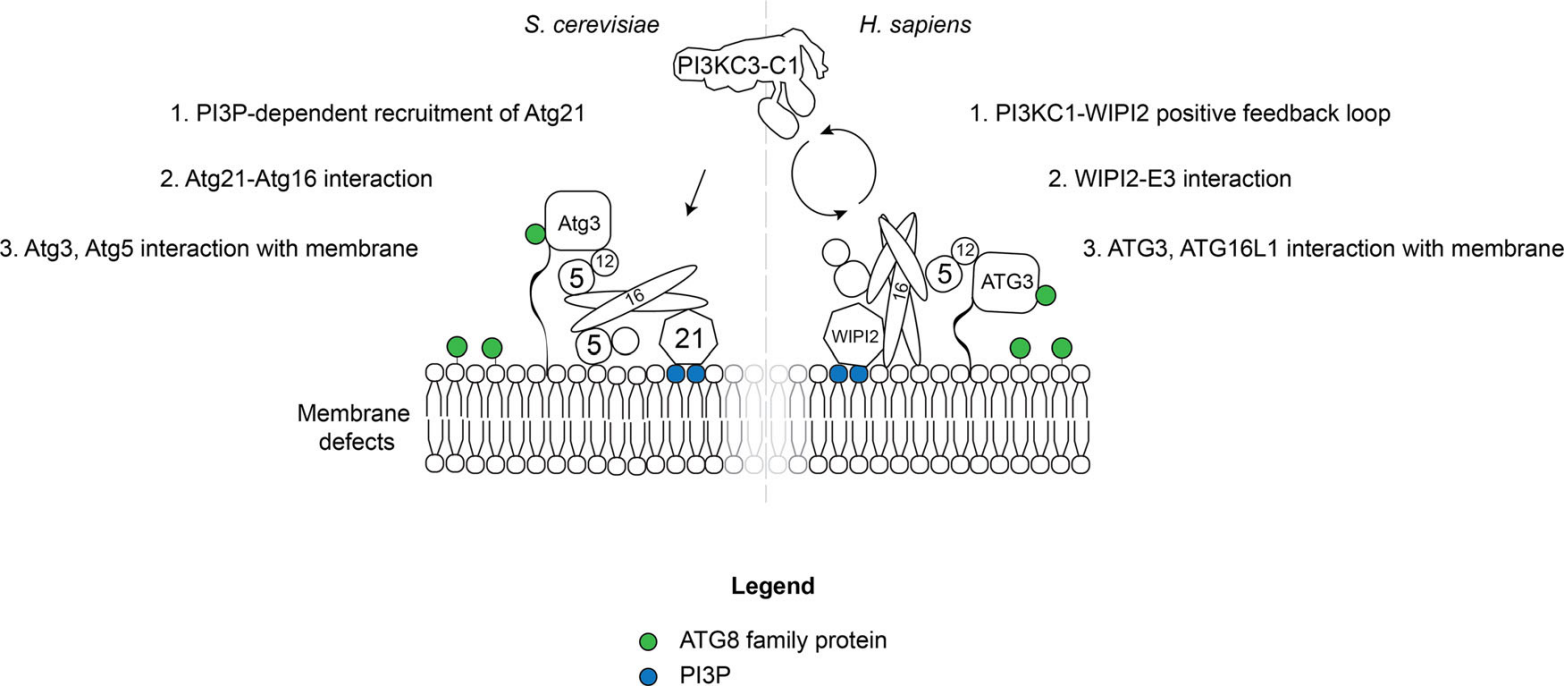
Activation and localization of the ATG8 conjugation machinery in macroautophagy. Schematic representation of the cascade that leads to ATG8 lipidation on the target membrane. In yeast and mammals PI3KC3-C1 activity is necessary to produce PI3P and to recruit PI3P-sensor proteins (Atg21 or WIPIs) to the pre-autophagosomal membrane. These proteins in turn bind the E3-like ligase via the ATG16 subunit and thereby recruit and activate the E3-like ligase activity. Correct membrane localization of the lipidation reaction is also mediated by the E2-like ATG3 that directly interacts with the ATG12 subunit of the E3 and its N-terminal amphipathic helix that inserts into the lipid bilayer (shown as black line). In yeast, ATG5 was shown to directly interact with poorly packed membranes, while in mammals ATG16L1 binds the membrane. Legend: the ATG8 protein family members are shown in green and the PI3P lipid is shown in blue.

In order to correctly target ATG8 lipidation, the substrate i.e. the membrane has to be permissive for the activity of ATG3 and the ATG12–ATG5–ATG16 complex needs to be present. In autophagy, the main determinant for the recruitment of the E3 is the ATG16 subunit (Fig. 3)^30,31,151^. In mammalian cells ATG16L1 is bound by the WIPI2 proteins, which in turn are recruited to the membrane by PI3P produced by the PI3KC3-C1^32^. In this manner, its recruitment is coupled to the activity of the lipid kinase complex. Additional targeting mechanisms also exists and likely work in conjunction with WIPI2 to robustly recruit and activate the E3 (Fig. 3)^34^. These mechanisms include the binding of ATG16L1 to FIP200^35,36^, which is a subunit of the ULK1/2 kinase complex^152^. In addition, ATG16L1 can directly bind to membranes that contain PI3P via two sites (Fig. 3)^23,34,38^. One site is located in the coiled-coil domain^38^ and the other site maps to a region C-terminal of this domain^23^. The extreme C-terminal WD domain of ATG16L1 is not required for its targeting in autophagy^153^. In combination, these targeting mechanisms increase the local concentration of the E3 and will thereby locally promote ATG8 lipidation. There may be additional activation mechanisms beyond the mere recruitment of the E3. For example, WIPI2 can directly activate the activity of the conjugation machinery (Fig. 3)^34^.

In yeast, the situation is similar as the interaction of Atg16 with the WIPI2 homolog Atg21 is important for the recruitment of Atg12–Atg5–Atg16 complex to the site of autophagosome formation^33^. However, notable differences exist. Atg21 interacts with the coiled-coil domain of Atg16 whereas WIPI2 binds to a motif C-terminal of this domain in ATG16L1^32,33^. The yeast the E3 can also bind to membranes but this interaction is mediated by the Atg5 subunit (Fig. 3)^39^. Furthermore, while Atg12–Atg5–Atg16 also interacts with the Atg1 kinase complex, the equivalent of the ULK1/2 complex, this interaction is mediated by the Atg12 subunit^37^.

Some specificity of the lipidation reaction may also be conferred by ATG3. Apart from the requirement for PE, membranes that display a high degree of membrane curvature, contain lipids with small headgroups or have membrane defects all facilitate ATG8 lipidation, likely by allowing insertion of amphipathic helix of ATG3 into the lipid bilayer^39,154,155^. Loosely, packed membranes containing a high percentage of lipids with unsaturated acyl chains appear to promote ATG8 conjugation at multiple levels^34^, consistent with their high abundance in autophagosomal membranes at least in yeast cells^156^.

**Fig. 4:**
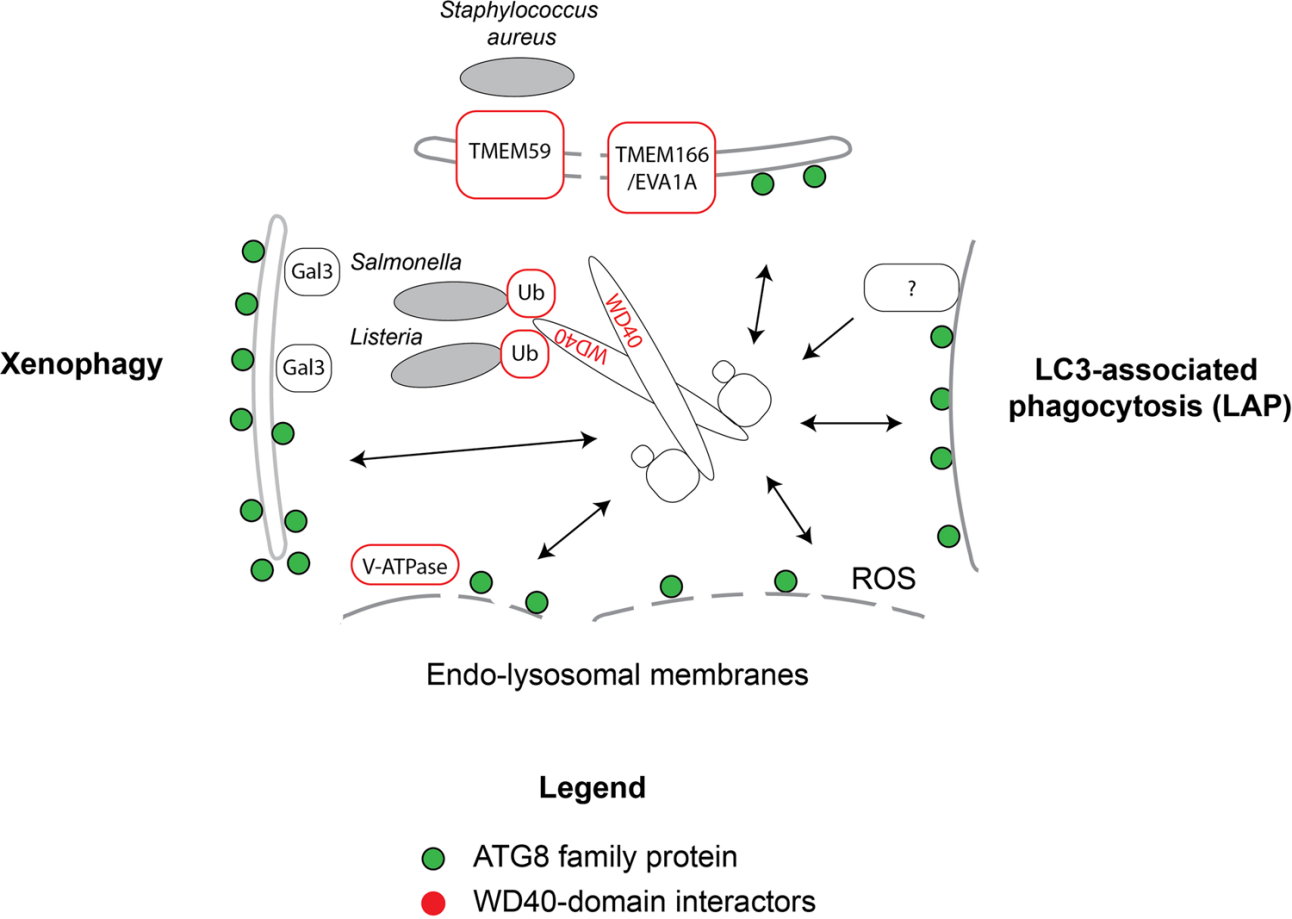
ATG8 conjugation to single membranes in non-autophagic events. Cartoon summarizing the recruitment of the ATG16L1 protein in ATG8 conjugation events that are not directly related to autophagy. The arrows indicate the recruitment of the ATG12–ATG5–ATG16L1 complex to single membranes. The factors recruiting the complex via the WD40 domain in ATG16L1 are highlighted in red.

In contrast to the situation in autophagy, where a wealth of knowledge about the targeting of ATG8 lipidation exists, little is known about how this reaction is targeted in autophagy-independent events. The main difference is that the C-terminal WD40 domain of ATG16L1 is dispensable for ATG8 lipidation in autophagy but essential for ATG8 conjugation in LAP and on endosomes and lysosomes (Fig. 4)^153,157^. Potential recruiting factors of ATG16L1 via the WD40 domain are the TMEM166/EVA1A^158^, the TMEM59 protein^159,160^, the V-ATPase^161^ and ubiquitin, which is present of vacuoles containing bacterial pathogens^162,163^ (Fig. 4). In addition, it was shown that galectin-3 recruits ATG16L1 via TRIM16 to damaged lysosomes^164,165^. The C-terminal membrane binding site in ATG16L1 was also shown to promote its membrane recruitment and ATG8 lipidation induced by lysosomal damage^23^. It is conceivable that a combination of a proteinaceous recruitment factor in conjunction with hydrophobic defects such as caused by membrane damage and acyl chain oxidization is the most effective way to recruit the ATG12–ATG5–ATG16L1 complex and thus to elicit ATG8 lipidation. Perhaps, the lipidated ATG8 proteins first recruit factors for membrane repair and if this fails, cargo receptors and the autophagy machinery that mediate autophagic engulfment of the damaged membrane structure.

In addition to the forward reaction catalyzed by the lipidation machinery, the reverse reaction in form of ATG4-mediated delipidation is a potential mechanism for the control of ATG8 protein density on the membrane. In fact, it was shown that in yeast inappropriately lipidated ATG8 is removed from membrane by Atg4^166^. Therefore, local activation of ATG8 lipidation in conjunction with inhibition of the ATG4-mediated reverse reaction may contribute to the control of the ATG8 densities on the membrane. This mechanism was already suggested during autophagosome biogenesis in yeast, where the Atg1 activity inhibits Atg4^65^.

## Conclusion

Considering the many factors that interact with ATG8 proteins in various pathway a perhaps obvious question is how the recruitment of the different factors is spatially and temporally regulated. For example, why are not all LIR containing autophagy factors recruited to the phagosomes during LAP and why are the cargo receptors and therefore the cargo attached to the inner but not the outer autophagosomal membrane? These questions are currently difficult to answer. Kinases that act together with antagonizing phosphatases are likely regulators. For example, TBK1 may locally increase the affinity of LIR motifs^167^ while phosphatases might quickly remove the phosphate. Furthermore, cargo receptors are frequently dimeric or even multimeric and will therefore select for surfaces with a high density of ATG8 proteins. They will also tend to have lower off-rates than monomeric ATG8 binders due to avidity effects. In contrast, monomeric proteins with higher affinities of their LIR motifs for ATG8 or higher on-rates might be preferentially recruited to membrane containing lower densities of ATG8. In addition, during autophagosome formation, the future outer membrane can make extensive and intimate contact with the ER and might therefore not be well accessible for cargo receptors^168,169^. Upon autophagosome completion a fraction of the ATG8 proteins is removed from the outer membrane, at least in yeast^170^. Therefore, the outer membrane may no longer be an attractive target for the receptors.

Since their original discovery the known roles of the ATG8 proteins have continuously expanded and more will certainly be discovered. This is reminiscent to ubiquitin itself, which was initially discovered as degradation tag in the ubiquitin-proteasome system and which is now implicated in virtually every complex cellular pathway^171–173^. ATG8 proteins are likely to keep surprising us with their cellular functions.
